# Supplementary material for: Cytokinetic abscission requires actin-dependent microtubule severing
Source: Nat Commun. 2024 Mar 2;15:1949. doi: 10.1038/s41467-024-46062-9 (PMC10908825; doi:10.1038/s41467-024-46062-9)
Supplement: Supplementary file 1 — Supplementary Information [file 41467_2024_46062_MOESM1_ESM.pdf]

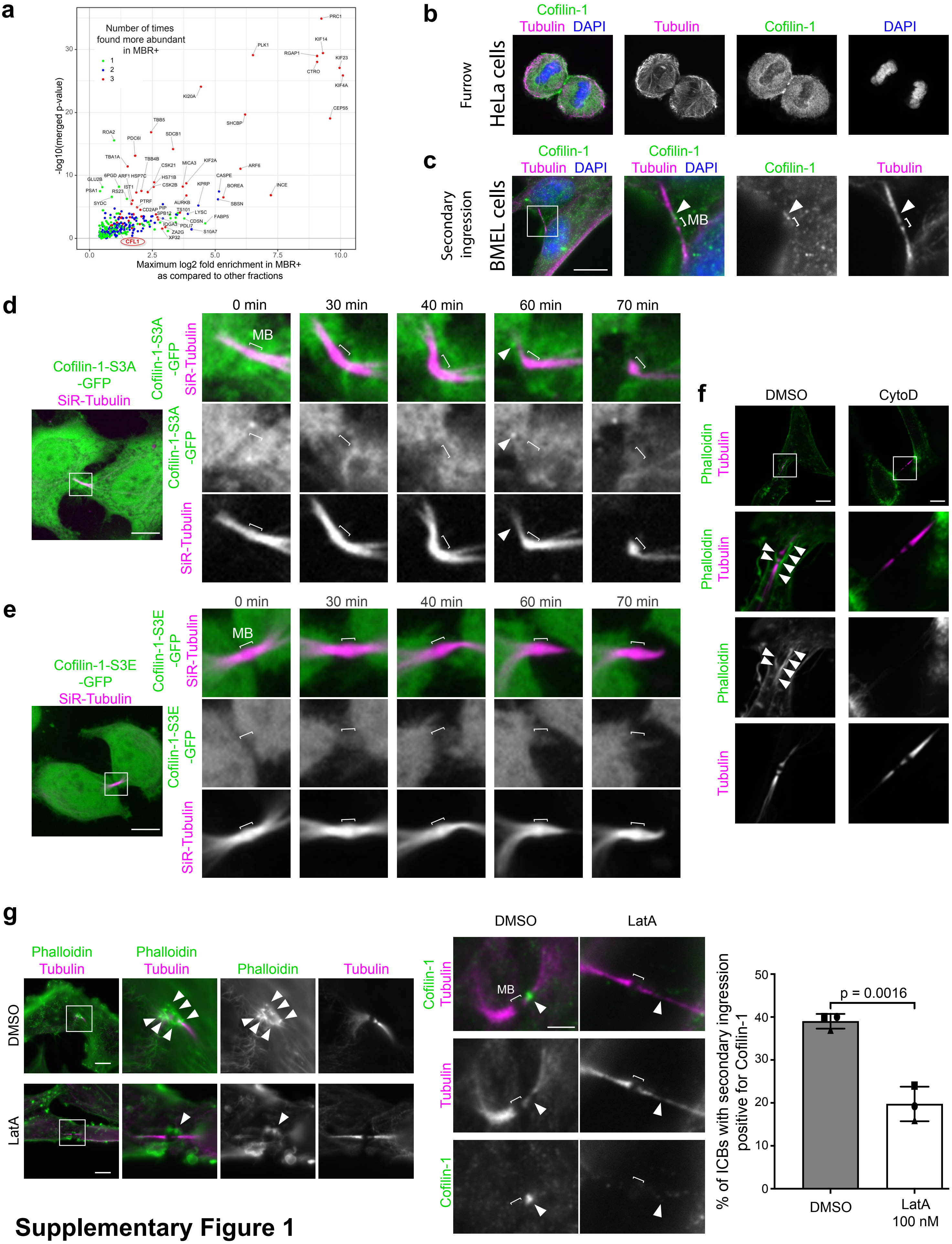

Supplementary Figure 1

Supplementary Figure 1: Transient recruitment of active, but not inactive Cofilin-1 at the secondary ingression shortly before the MT cut.

(a) Merged volcano plot of the mass spectrometry analysis showing the enriched Flemmingsome of HeLa cells described in ref.[21]. Briefly, midbody remnants (MBR) from HeLa cells expressing the midbody marker MKLP2-GFP were purified by flow cytometry (MBR+ fraction). The plot shows the -log10(merged p-value) (y-axis) in function of the corresponding maximum log2(fold change) measured between MBR+ fractions and the other fractions (total cell lysates, MBRs enriched by centrifugation or GFP-negative fraction) (x-axis). Cofilin-1 (= CFL1, circle) is significantly enriched by approximately 2-folds in MBRs.

(b) Staining of endogenous Cofilin-1 and Tubulin in HeLa cells showing the recruitment of Cofilin-1 at the cytokinetic furrow. Scale bar = 10 μm. This experiment was repeated at least three times independently with similar results.

(c) Staining of endogenous Cofilin-1 and Tubulin in BMEL cells. Arrowhead: Cofilin-1 at the secondary ingression. Scale bar = 10 μm. This experiment was repeated two times independently with similar results.

(d) Snapshots of a spinning disk confocal microscopy movie of HeLa cells transiently transfected with Cofilin-1-S3A-GFP and incubated with fluorescent SiR-Tubulin. Arrowhead: transient recruitment of Cofilin-1 at the secondary ingression. Scale bar = 10 μm.

(e) Snapshots of a spinning disk confocal microscopy movie of HeLa cells transiently transfected with Cofilin-1-S3E-GFP and incubated with fluorescent SiR-Tubulin. Scale bar = 10 μm. Note that Cofilin-1-S3E-GFP is not recruited at the secondary ingression.

(f) Staining of endogenous Tubulin and F-actin (labelled with fluorescent phalloidin) in HeLa cells treated with either DMSO or 0.4 μM Cytochalasin D (CytoD) for 1 hour prior to fixation. Arrowheads: F-actin localization at the ICB. Scale bars = 10 μm.

(g) Left: Staining of endogenous Tubulin and F-actin (labelled with fluorescent phalloidin) in HeLa cells treated with either DMSO or 100 nM Latrunculin-A (LatA) for 1 hour prior to fixation. Arrowheads: F-actin localization at the ICB. Scale bars = 5 μm. Middle: Staining of endogenous Cofilin-1 and Tubulin in late ICBs with secondary ingression (arrowheads) in cells treated as indicated above. Scale bars = 2 μm. Right: Percentage of ICBs with secondary ingression positive for Cofilin-1. Mean $\pm$ SD, $n \geq 25$ cells per condition, N= 3 independent experiments. Two-tailed unpaired Student's t test.

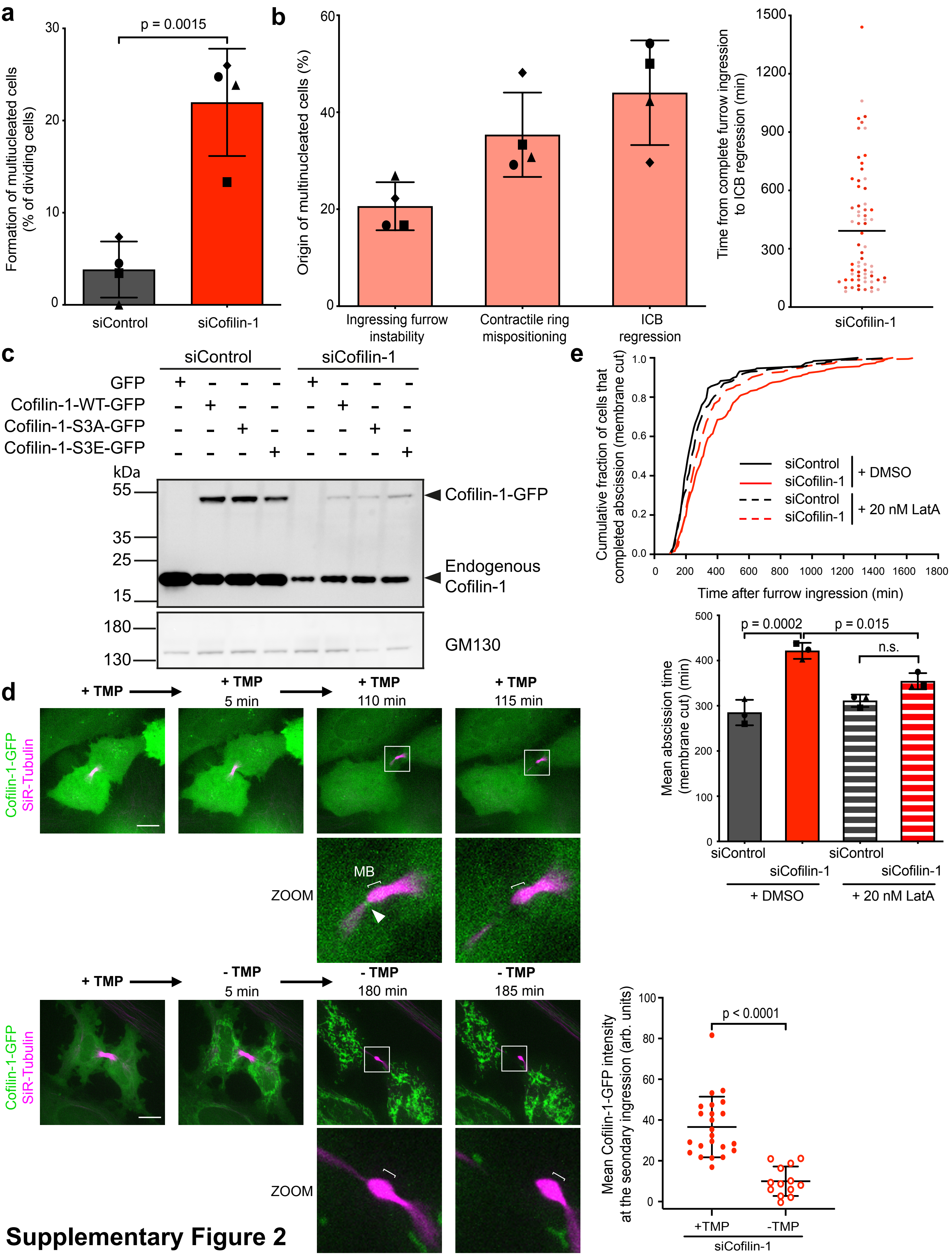

Supplementary Figure 2

Supplementary Figure 2: Defective abscission upon Cofilin-1 depletion can lead to the appearance of multinucleated cells.

(a) Percentage of cell divisions recorded by live cell imaging giving rise to multinucleated cells upon treatment with either Control or Cofilin-1 siRNAs. Mean ± SD, n≥ 59 cells per condition, N= 4 independent experiments. Two-tailed unpaired Student's t test.

(b) Left: Proportion of multinucleated cells observed in (a) upon Cofilin-1 depletion arising from ingressing furrow instability, contractile ring mispositioning or ICB regression. Mean ± SD, n= 12-27 cells per experiment, N= 4 independent experiments. Right: time (min) from complete furrow ingression to ICB regression in cells depleted for Cofilin-1. Mean value and all data points (n= 66 cells) from 4 independent experiments (in different colors) are represented.

(c) Lysates from HeLa cells treated with either Control or Cofilin-1 siRNAs and transiently transfected with either GFP or siRNA-resistant Cofilin-1-GFP constructs, as indicated, were blotted with anti-Cofilin-1 and anti-GM130 antibodies (loading control). Note that siCofilin-1 treated cells were less efficiently transfected than control cells. This experiment was repeated two times independently with similar results.

(d) Left: Snapshots of a spinning disk confocal microscopy movie of cells stably co-expressing Cofilin-1-GFP and the Tom20-anti-GFP nanobody-SNAP-DHFR fusion protein (labelled with SiR-SNAP) upon Cofilin-1 depletion either when TMP is present (upper panels) or upon TMP removal (lower panels), with a zoom on a secondary ingression. Arrowhead: Cofilin-1-GFP at the secondary ingression. Scale bar = 10 μm. Right: Quantification of Cofilin-1-GFP mean fluorescence intensity at the secondary ingression with or without TMP. Mean± SD , n = 13 (-TMP) or 23 (+TMP) cells per condition. Two-sided Mann Whitney test.

(e) Top: same analysis as in main Fig. 2b for cells treated with either Control or Cofilin-1 siRNAs and incubated with either DMSO or 20 nM Latrunculin A (LatA) (n ≥ 184 cells per condition from N= 3 independent experiments). Bottom: Mean abscission time (min) ± SD in indicated cells, n ≥ 61 cells per condition, N= 3 independent experiments. One-way ANOVA with Tukey's multiple comparisons test. n.s. = non-significant ($p > 0.05$).

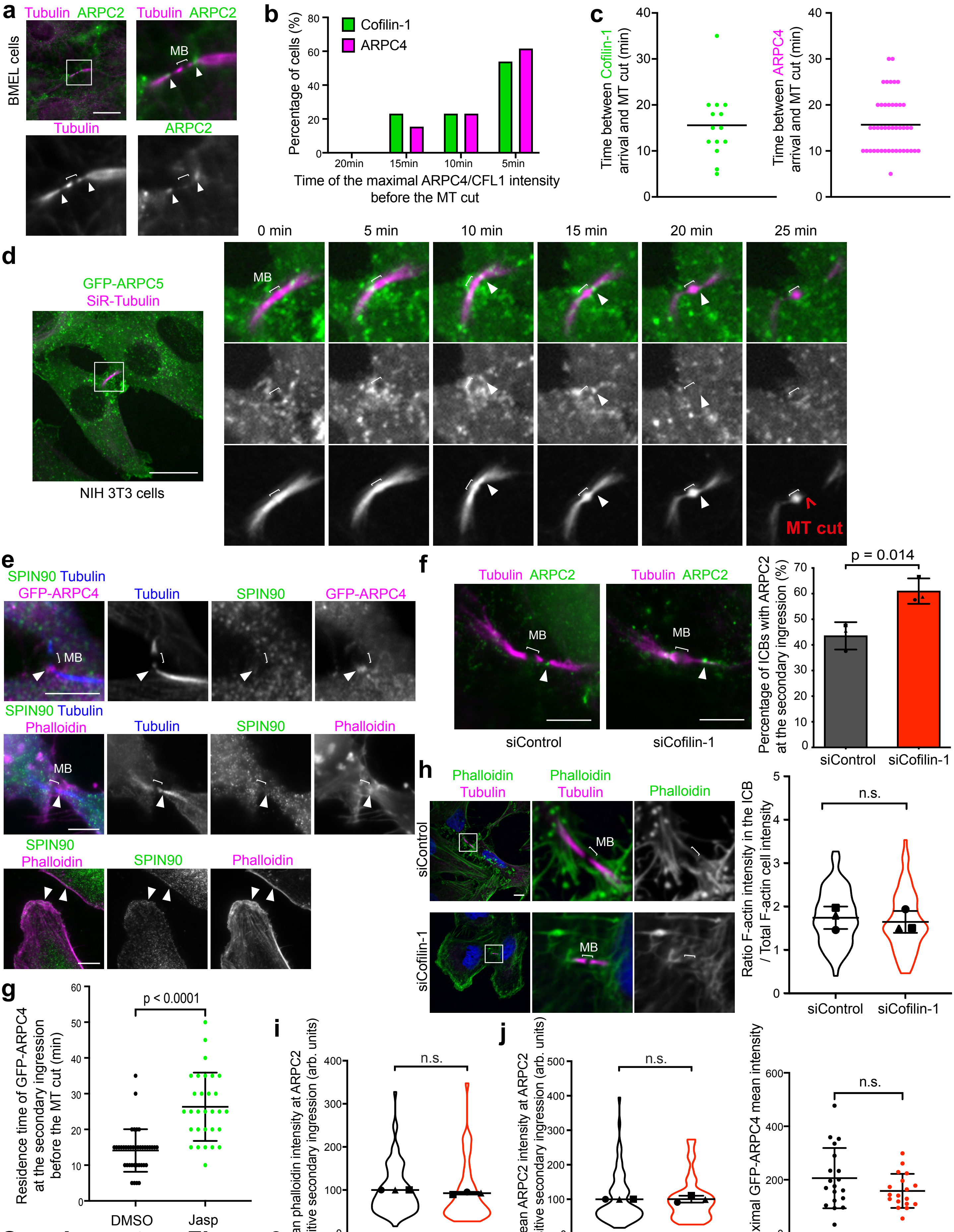

Supplementary Figure 3

Supplementary Figure 3: The transient recruitment of Arp2/3 at the secondary ingression is regulated by Cofilin-1.

(a) Staining of endogenous ARPC2 and Tubulin in late ICBs with secondary ingression of mouse BMEL cells. Arrowhead: ARPC2 at the secondary ingression. Scale bar = 5 μm. This experiment was repeated two times independently with similar results.

(b) Histogram representing the repartition of the time of the maximal Cofilin-1-GFP/mApple-ARPC4 intensity at the secondary ingression before the MT cut, as described in main Fig. 3b. n= 13 cells.

(c) Left: Time (min) between Cofilin-1-GFP detected at the secondary ingression and the MT cut in cells described in Fig. 1c and recorded by spinning-disk confocal microscopy. n= 14 cells. The black line corresponds to the mean value. Right: Time (min) between mApple-ARPC4 detected at the secondary ingression and the MT cut in cells described in Fig. 6b and recorded by spinning-disk confocal microscopy. n= 44 cells. The black line corresponds to the mean value.

(d) Snapshots of a spinning disk confocal microscopy movie of mouse NIH3T3 cells stably expressing GFP-ARPC5 and incubated with fluorescent SiR-Tubulin. Arrowhead: ARPC5 localization at the secondary ingression. Scale bar = 10 μm.

(e) Top and middle: Staining of endogenous SPIN90, Tubulin and GFP-ARPC4 or F-actin (labelled with fluorescent phalloidin) in ICBs with secondary ingression, as indicated. Note the absence of SPIN90 at the ARPC4- or F-actin-positive pool present at the secondary ingression (arrowhead). Scale bar = 5 μm. Bottom: Staining of endogenous SPIN90 and F-actin (labelled with fluorescent phalloidin) showing partial colocalization at the cell cortex (arrowheads), as expected. Scale bar = 10 μm. This experiment was repeated two times independently with similar results.

(f) Left: Staining of endogenous ARPC2 and Tubulin in ICBs with secondary ingression upon treatment with either Control or Cofilin-1 siRNAs. Arrowhead: ARPC2 at the secondary ingression. Scale bar = 5 μm. Right: Percentage of ICBs with ARPC2 at the secondary ingression in indicated cells. Mean ± SD, n ≥ 32 cells per condition, N= 3 independent experiments. Two-tailed unpaired Student's t test.

(g) The time elapsed between GFP-ARPC4 appearance at the secondary ingression and the MT cut was measured as described in main Fig. 3d in non-depleted cells treated with either DMSO

or 50 nM of Jasplakinolide (Jasp). Mean ± SD in indicated cells, n= 39 (DMSO) or 30 (Jasp) cells per condition from N= 3 independent experiments. Two-sided Mann-Whitney test.

(h) Left: Staining of endogenous Tubulin and F-actin (labelled with fluorescent phalloidin) in HeLa cells treated with either Control or Cofilin-1 siRNAs. Scale bar = 10 μm. Right: Ratio of the mean phalloidin fluorescence intensity in the ICB and the mean phalloidin fluorescence intensity of the entire cell. Mean ± SD in indicated cells, n ≥ 20 cells per condition, N= 3 independent experiments (violin plots). Two-tailed unpaired Student's t test. n.s. = non-significant ($p > 0.05$).

(i) Mean phalloidin fluorescence intensity at ARPC2-positive secondary ingression. Mean ± SD, n = 15-30 cells per condition, N= 3 independent experiments (violin plots). Intensities are normalized in each experiment to the mean intensity of siControl-treated cells. Two-tailed unpaired Student's t test with Welch's correction. n.s. = non-significant ($p > 0.05$).

(j) Left: Mean endogenous ARPC2 fluorescence intensity at ARPC2-positive secondary ingression. Mean ± SD, n = 15-30 cells per condition, N= 3 independent experiments (violin plots). Intensities are normalized in each experiment to the mean intensity of siControl-treated cells. Two-tailed unpaired Student's t test with Welch's correction. n.s. = non-significant ($p > 0.05$). Right: Maximal measured mean GFP-ARPC4 fluorescence intensity at ARPC4-positive secondary ingression in movies of GFP-ARPC4 expressing cells (as described in main Fig. 3d). Mean ± SD, n ≥ 18 cells per condition from N= 4 independent experiments. Two-sided Mann-Whitney test. n.s. = non-significant ($p > 0.05$).

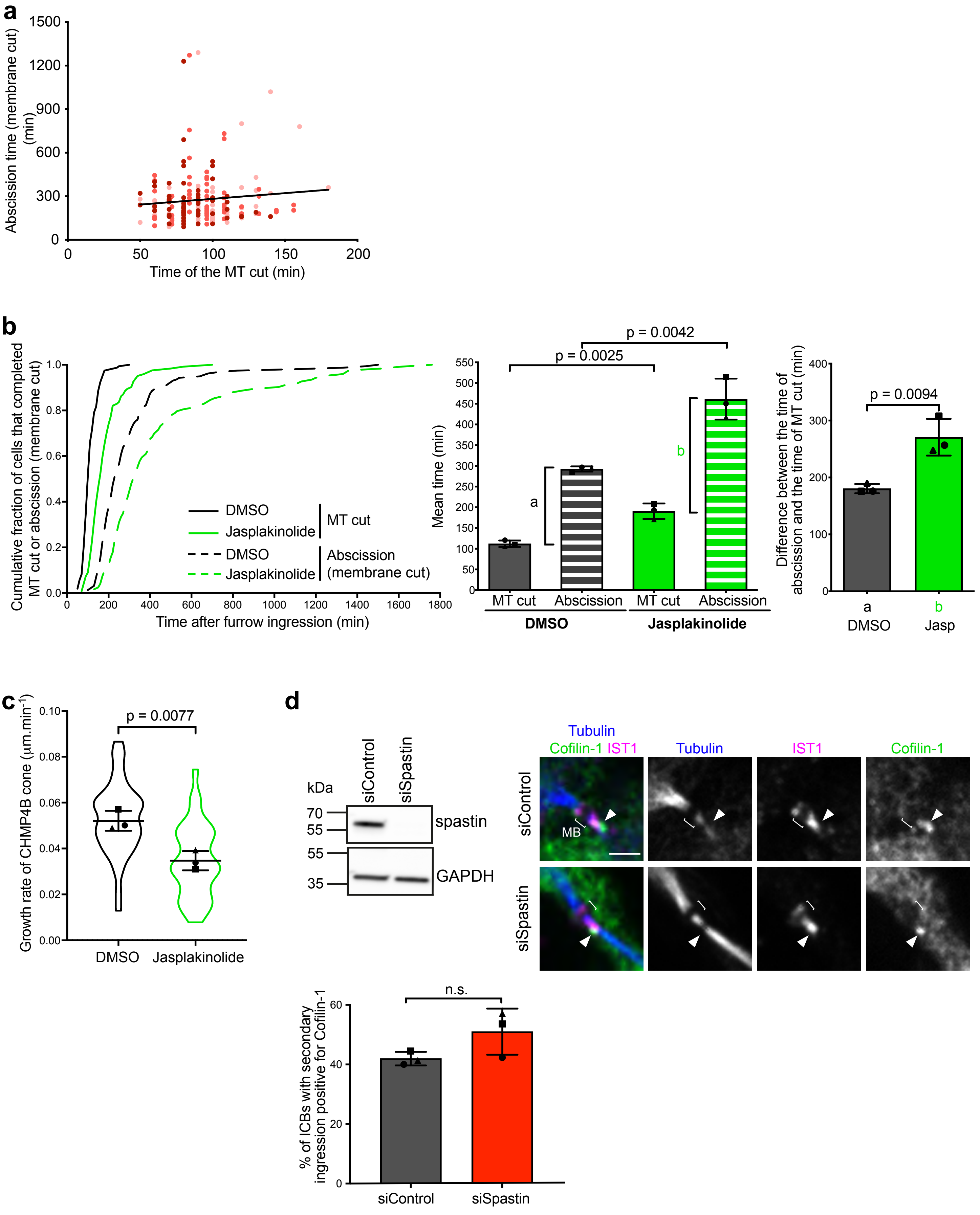

Supplementary Figure 4

Supplementary Figure 4: Time of the MT cut as a function of the abscission time. Effect of Jasplakinolide on MT cut and abscission. Effect of spastin depletion on Arp2/3 at the secondary ingression.

(a) Time (min) of the MT cut as a function of the abscission time (membrane cut) in cells treated with Control siRNAs and presented in main Fig. 4b. Each dot corresponds to one cell division. n= 190 cells from 3 independent experiments (in different colors). Black line: linear regression fitted to the raw data. Spearman r correlation test, p= 0.47.

(b) Left: Cumulative distribution of the fraction of cells that completed MT cut (solid curves) and abscission (dashed curves) as function of time after complete furrow ingression, in cells treated with either DMSO or 50 nM Jasplakinolide (n $\geq$ 123 cells per condition from N= 3 independent experiments). MT cut and abscission timings were determined in the same cells using the fluorescent SiR-Tubulin channel and the phase contrast channel, respectively. Middle: Mean MT cut and abscission time (min) $\pm$ SD in indicated cells, n = 25-66 cells per condition, N= 3 independent experiments. Two-tailed unpaired Student's t test. a and b represent the time difference between the abscission and the MT cut in DMSO and Jasplakinolide-treated cells, respectively. Right: a and b values defined in the middle panel have been represented. Two-tailed unpaired Student's t test.

(c) Quantification of the growth rate of CHMP4B cone in HeLa cells stably expressing CHMP4B-GFP after treatment with either DMSO or 50 nM Jasplakinolide. Mean $\pm$ SD, n= 9-18 cells per condition, N= 3 independent experiments (violin plots). Two-tailed unpaired Student's t test.

(d) Top left: Lysates of HeLa cells treated with either control or spastin siRNAs were blotted for endogenous spastin and GAPDH (loading control). This experiment was repeated two times independently with similar results. Top Right: Staining of endogenous Cofilin-1, Tubulin and IST1 in ICBs with secondary ingression upon treatment with either Control or spastin siRNAs. Arrowhead: Cofilin-1 at the secondary ingression. Scale bar = 2 µm. Bottom left: Percentage of ICBs with Cofilin-1 at the secondary ingression in indicated cells. Mean $\pm$ SD, n $\geq$ 25 cells per condition, N= 3 independent experiments. Two-tailed unpaired Student's t test. n.s. = non-significant (p > 0.05).

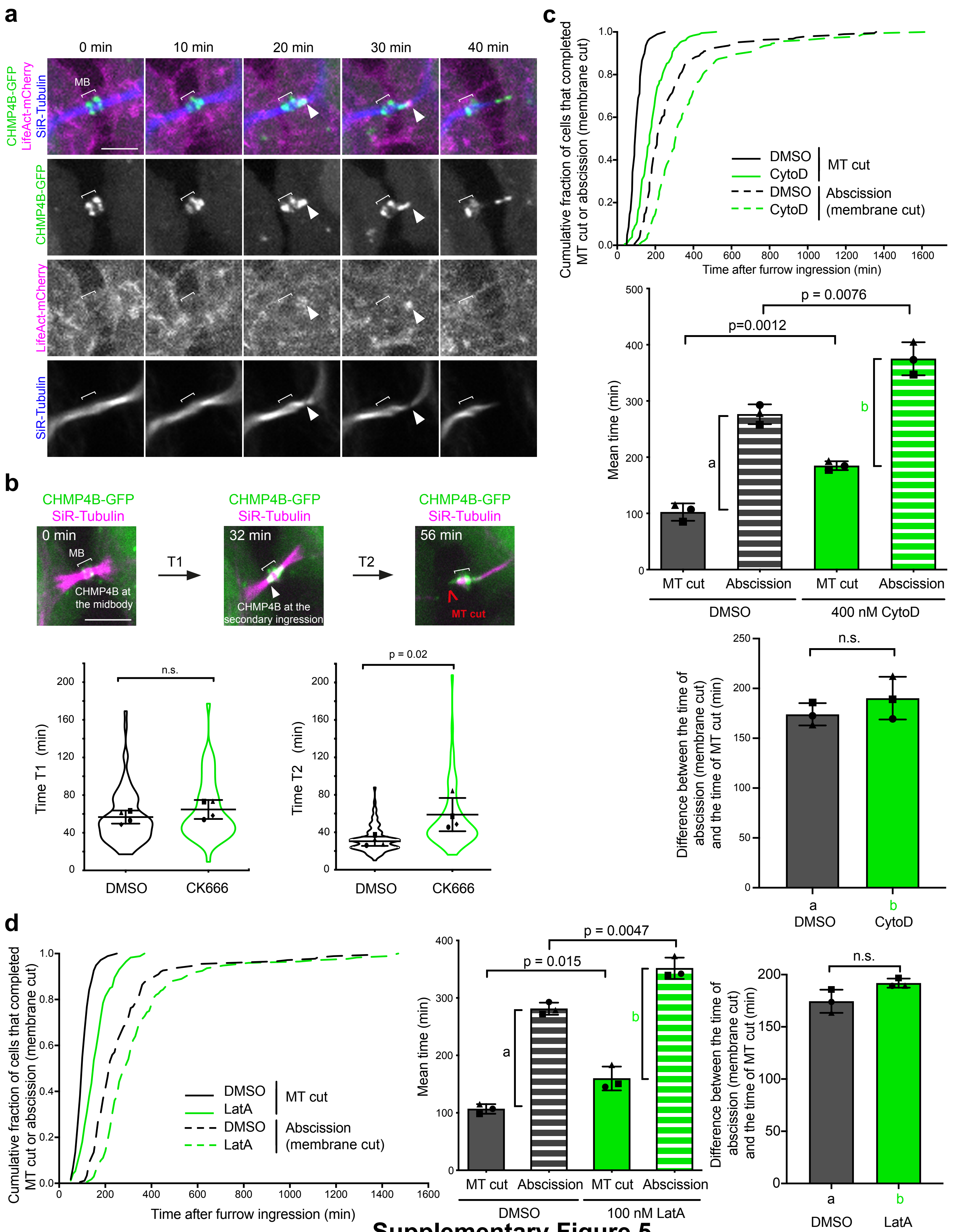

Supplementary Figure 5

Supplementary Figure 5: A pool of F-actin localizes at the tip of the ESCRT-III cone shortly before the MT cut. F-actin depolymerization delays the MT cut.

(a) Snapshots of a spinning disk confocal microscopy movie of cells stably co-expressing CHMP4B-GFP and LifeAct-mCherry and incubated with fluorescent SiR-Tubulin Arrowheads: transient F-actin localization at the tip of the CHMP4B cone. Scale bar = 10 μm.

(b) Top: Snapshots of a spinning disk confocal microscopy movie of cells stably expressing CHMP4B-GFP and incubated with fluorescent SiR-Tubulin as in Fig. 5b. T1: time from CHMP4B-GFP recruitment at the midbody (t = 0 min) to appearance at the secondary ingression (arrowhead); T2: time from CHMP4B-GFP appearance at the secondary ingression to the MT cut. Scale bar = 5 μm. Bottom: Mean T1 and T2 (min) $\pm$ SD in indicated cells, n= 4-45 cells per condition, N= 4 independent experiments (violin plots). Two-tailed unpaired Student's t test with Welch's correction. n.s. = non-significant ($p > 0.05$).

(c) Top: Cumulative distribution of the fraction of cells that completed MT cut (solid curves) and abscission (dashed curves) as function of time after complete furrow ingression, in cells treated with either DMSO or 0.4 μM Cytochalasin D (CytoD) (n $\geq$ 182 cells per condition from N= 3 independent experiments). MT cut and abscission timings were determined in the same cells using the fluorescent SiR-Tubulin channel and the phase contrast channel, respectively. Middle: Mean MT cut and abscission time (min) $\pm$ SD in indicated cells, n = 60-65 cells per condition, N= 3 independent experiments. Two-tailed unpaired Student's t test. a and b represent the time difference between the abscission and the MT cut in DMSO and CytoD-treated cells, respectively. Bottom: a and b values defined in the left panel have been represented. Two-tailed unpaired Student's t test. n.s. = non-significant ($p > 0.05$).

(d) Left: Cumulative distribution of the fraction of cells that completed MT cut (solid curves) and abscission (dashed curves) as function of time after complete furrow ingression, in cells treated with either DMSO or 100 nM Latrunculin A (LatA) (n $\geq$ 172 cells per condition from N= 3 independent experiments). MT cut and abscission timings were determined in the same cells using the fluorescent SiR-Tubulin channel and the phase contrast channel, respectively. Middle: Mean MT cut and abscission time (min) $\pm$ SD in indicated cells, n = 51-65 cells per condition, N= 3 independent experiments. Two-tailed unpaired Student's t test. a and b represent the time difference between the abscission and the MT cut in DMSO and LatA-

treated cells, respectively. Right: a and b values defined in the left panel have been represented. Two-tailed unpaired Student’s t test.

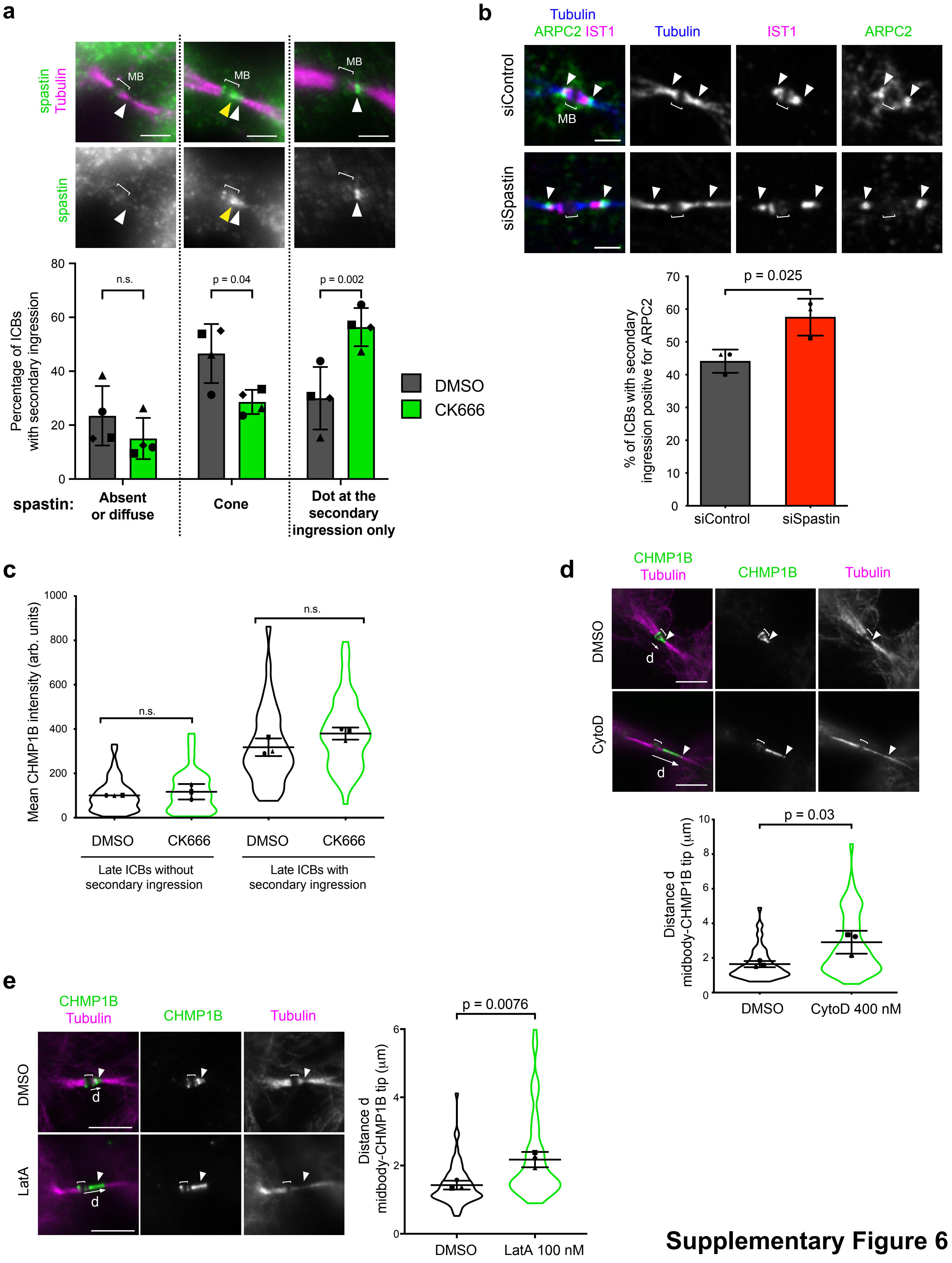

Supplementary Figure 6

Supplementary Figure 6: F-actin depolymerization leads to continuous extension of the ESCRT-III cone.

(a) Top: Staining of endogenous spastin and Tubulin in ICBs with secondary ingressions. Spastin patterns can be classified in 3 categories: absent/diffuse, cone, dot at the secondary ingression only. Arrowhead: secondary ingression. Yellow arrowhead: spastin cone at the midbody side. Scale bars: 2 µm. Bottom: Percentage of ICBs with secondary ingression displaying each category of spastin localization in cells incubated with either DMSO or 200 µM CK666 for 2 hours. Mean ± SD, n= 13-21 cells per condition, N= 4 independent experiments. Two-way ANOVA with Sidak's multiple comparisons test. Adjusted p-values are represented.

(b) Top: Staining of endogenous ARPC2, Tubulin and IST1 in ICBs with secondary ingression upon treatment with either control or spastin siRNAs. Arrowhead: ARPC2 at the secondary ingression. Scale bar = 2 µm. Bottom: Percentage of ICBs with ARPC2 at the secondary ingression in indicated cells. Mean ± SD, n ≥ 25 cells per condition, N= 3 independent experiments. Two-tailed unpaired Student's t test.

(c) Mean fluorescence intensity of CHMP1B (arbitrary units) at the midbody (late ICBs without secondary ingression) or at the midbody + secondary ingression (late ICBs with secondary ingression) in cells treated with either DMSO or 200 µM CK666 for 2 hours. Mean ± SD, n= 8-21 cells per condition, N= 3 independent experiments (violin plots). Intensities are normalized in each experiment to the mean intensity of DMSO-treated cells in late ICB without secondary ingression. One-way ANOVA with Tukey's multiple comparisons test. n.s. = non-significant ($p > 0.05$).

(d) Top: Staining of endogenous CHMP1B and Tubulin in cells treated with either DMSO or 0.4 µM Cytochalasin D (CytoD) for 1 hour prior to fixation. Scale bars = 5 µm. Arrowhead: secondary ingression. Bottom: Distance d (µm) between the midbody center and the tip of the CHMP1B cone in indicated cells. Mean ± SD, n= 9-27 ICBs with a secondary ingression per condition, N= 3 independent experiments (violin plots). Two-tailed unpaired Student's t test.

(e) Left: Staining of endogenous CHMP1B and Tubulin in cells treated with either DMSO or 100 nM Latrunculin A (LatA) for 1 hour prior to fixation. Scale bars = 5 µm. Arrowhead: secondary ingression. Right: Distance d (µm) between the midbody center and the tip of the CHMP1B cone in indicated cells. Mean ± SD, n= 21-25 ICBs with a secondary ingression per condition, N= 3 independent experiments (violin plots). Two-tailed unpaired Student's t test.

Fig. 2a

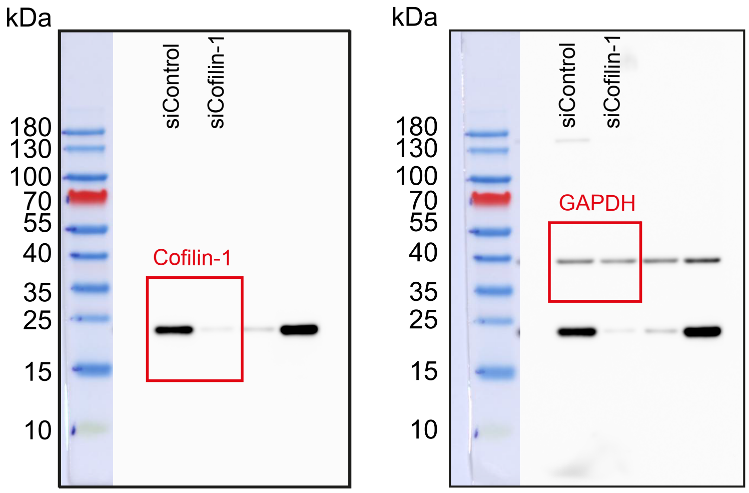

Supplementary Fig. 2c

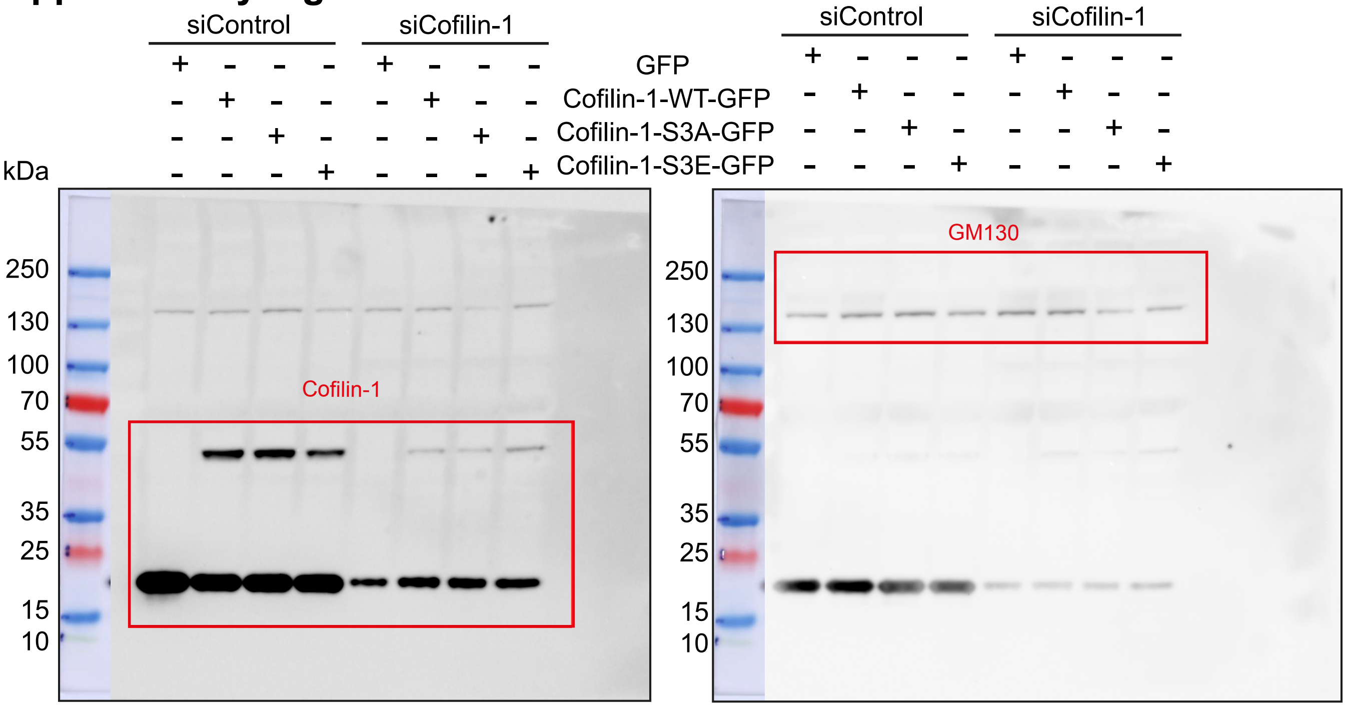

Supplementary Fig. 4d

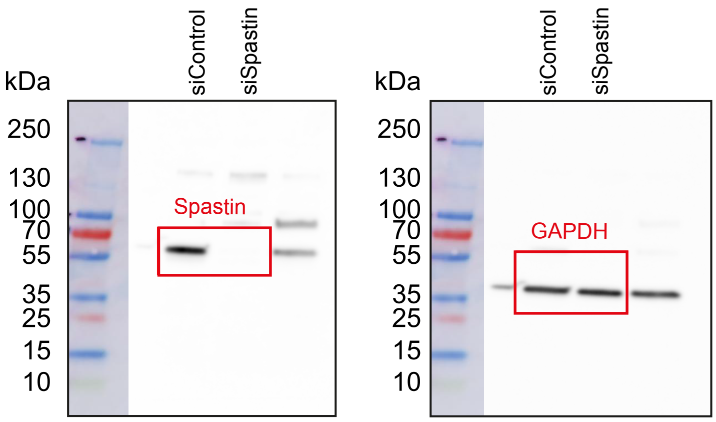

Supplementary Figure 7

Supplementary Figure 7: Uncropped western blots presented in Figure 2a, Supplementary Figures 2c and 4d.

Figure 2a: Left: the membrane was incubated with the anti-Cofilin-1 antibody only. Right: the membrane was next incubated with the anti-GAPDH antibody only.

Supplementary Figures 2c: Left: the membrane was incubated with anti-Cofilin-1 and anti-GM130 antibodies. Right: the membrane was next re-incubated with the anti-GM130 antibody only.

Supplementary Figures 4d: Left: the membrane was incubated with the anti-spastin antibody only. Right: the membrane was next incubated with the anti-GAPDH antibody only.

Antibodies

Target protein	Company	Host	Reference/clone	dilution WB	dilution IF	fixation IF
Cofilin-1	Cell Signaling technology	Rabbit	#5175		1/250	MetOH
Cofilin-1	Proteintech	Mouse	66057-1-Ig	1/10000	1/200	MetOH
Tubulin	Institut Curie	Human	F2C-hFc2, VHHD5-hFc1, C3B9-hFc		1/100	PFA, MetOH
ARPC2	Millipore	Rabbit	07-227		1/200	PFA
CHMP4B	Proteintech	Rabbit	13683-1-AP		1/500	MetOH
CHMP1B	Proteintech	Rabbit	14639-1-AP		1/500	MetOH
CHMP1B	Santa Cruz	Mouse	sc-514013 (D-10)		1/100	MetOH
GAPDH	Proteintech	Mouse	60004-1-Ig (1E6D9)	1/40 000		
Spastin	Proteintech	Rabbit	22792-1-AP	1/2 000	1/200	MetOH
IST1	Proteintech	Mouse	66989-1-Ig (1E12A7)		1/500	MetOH
SPIN90	Proteintech	Rabbit	11367-1-AP		1/200	PFA
GM130	BD transduction	Mouse	610822	1/500		

Supplementary Table 1
