# Supplementary material for: Cytokinetic abscission requires actin-dependent microtubule severing
Source: Nat Commun. 2024 Mar 2;15:1949. doi: 10.1038/s41467-024-46062-9 (PMC10908825; doi:10.1038/s41467-024-46062-9)
Supplement: Supplementary file 2 — Description of Additional Supplementary Files [file 41467_2024_46062_MOESM2_ESM.pdf]

Description of Additional Supplementary Files

Supplementary Movie Legends

Supplementary Movie 1: Spinning disk confocal microscopy movie of cells stably co-expressing Cofilin-1-GFP (green) and LifeAct-mCherry (magenta) and incubated with fluorescent SiR-Tubulin (blue). White arrowheads: Cofilin-1 and LifeAct co-localization at the secondary ingression. Time interval: 7 min. Scale bar = 5 μm.

Supplementary Movie 2: Spinning disk confocal microscopy movie of cells stably co-expressing Cofilin-1-GFP (green) and mApple-ARPC4 (magenta) and incubated with fluorescent SiR-Tubulin (blue). White arrowheads: Cofilin-1 and ARPC4 co-localization at the secondary ingression. Red arrowhead: Microtubule cut (t = 15 min). Time interval: 5 min. Scale bar = 5 μm.

Supplementary Movie 3: Widefield microscopy movie of HeLa cells incubated with fluorescent SiR-Tubulin (red) and recorded both in phase contrast and in fluorescence. Time 0 corresponds to the time of complete furrow ingression. Red arrowhead: Microtubule cut (t = 70 min). Yellow arrowhead: Membrane cut = abscission (t = 170 min). Yellow double headed arrows: orientation of the midbody visualized by SiR-Tubulin. Note the rotation of the midbody at the time of the membrane cut. Time interval: 10 min. Scale bar = 10 μm.

Supplementary Movie 4: Spinning disk confocal microscopy movie of cells stably expressing inducible GFP-spastin M87 (green) and incubated with fluorescent SiRTubulin (magenta), either in the presence of DMSO (left) or 200 μM CK666 (right). White arrowheads: spastin at secondary ingressions. Red arrowhead: Microtubule cut. Time interval: 3 min. Scale bar = 5 μm.

Supplementary Movie 5: Spinning disk confocal microscopy movie of cells stably co-expressing CHMP4B-GFP (green) and mApple-ARPC4 (magenta) and incubated with fluorescent SiR-Tubulin (blue), either in the presence of DMSO (left) or 200 μM CK666 (right) added between t= 0 and t= 5 min. White arrowheads: ARPC4 localization at the tip of the CHMP4B cone. Red arrowhead: Microtubule cut. Time interval: 5 min. Scale bar = 2 μm
